# Supplementary material for: OsMADS58 Stabilizes Gene Regulatory Circuits during Rice Stamen Development
Source: Plants (Basel). 2022 Oct 28;11(21):2899. doi: 10.3390/plants11212899 (PMC9658454; doi:10.3390/plants11212899)
Supplement: Supplementary file 1 [file plants-11-02899-s001.zip › Supplementary Figures.pdf]

SUPPLEMENTARY FIGURES

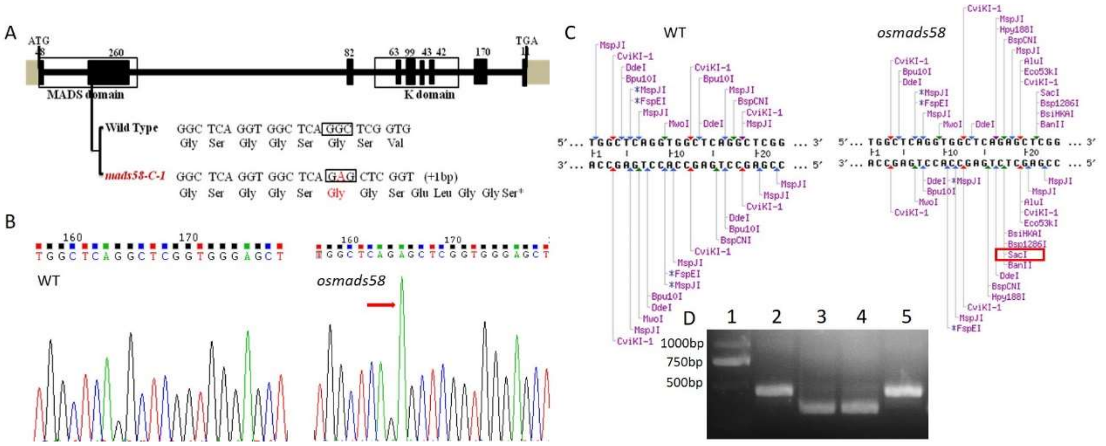

Supplementary Figure S1. Null mutation of *OsMADS58* is created by CRISPR technology (insert A transgenic line).

(A) Schematic diagram of the CRISPR constructs designed for *OsMADS58*. Target sequence is located in the second exon. Red "A" represents the inserted nucleotides on the gene in the mutant. The single nucleotide insertion caused a frameshift and a premature translation termination in *osmads58* mutant. This mutant was used for subsequent phenotypic observations and RNA-seq. (B) The *osmads58* mutation caused by CRISPR were detected using sequencing. The red arrow represents the insertion of a single nucleotide A in the homozygous mutant. (C) The target sequence restriction site display. The homozygous mutant inserted the nucleotide A can be recognized by SacI restriction endonuclease. (D) The *osmads58* homozygous mutant were detected using enzyme digestion. 1. marker; 2 and 5, *OsMADS58* gene seqence in wild-type were digested by SacI; 3 and 4, *OsMADS58* gene seqence in *osmads58* homozygous mutant were digested by SacI.

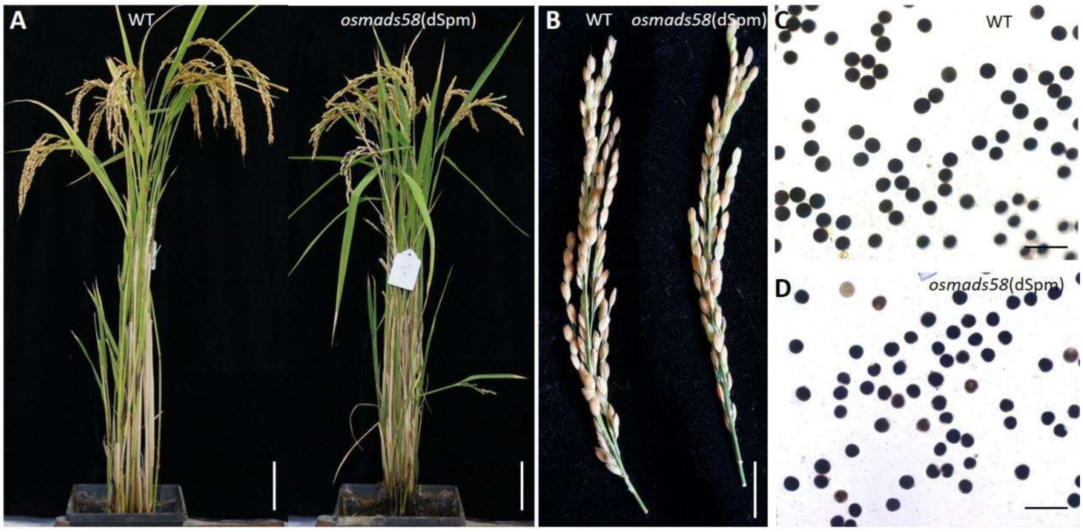

Supplementary Figure S2. The *osmads58* dSpm line exhibits normal fertility.

(A) Comparison of wild-type (WT) and *osmads58* dSpm line plants after seed maturation. Bars, 10 cm. (B) Panicles in wild-type and *osmads58* dSpm line plants. Bars, 1cm. (C and D) Wild-type (C) and *osmads58* dSpm line (D) pollen grains were stained by I_2-KI solution. Bars, 200 μm.

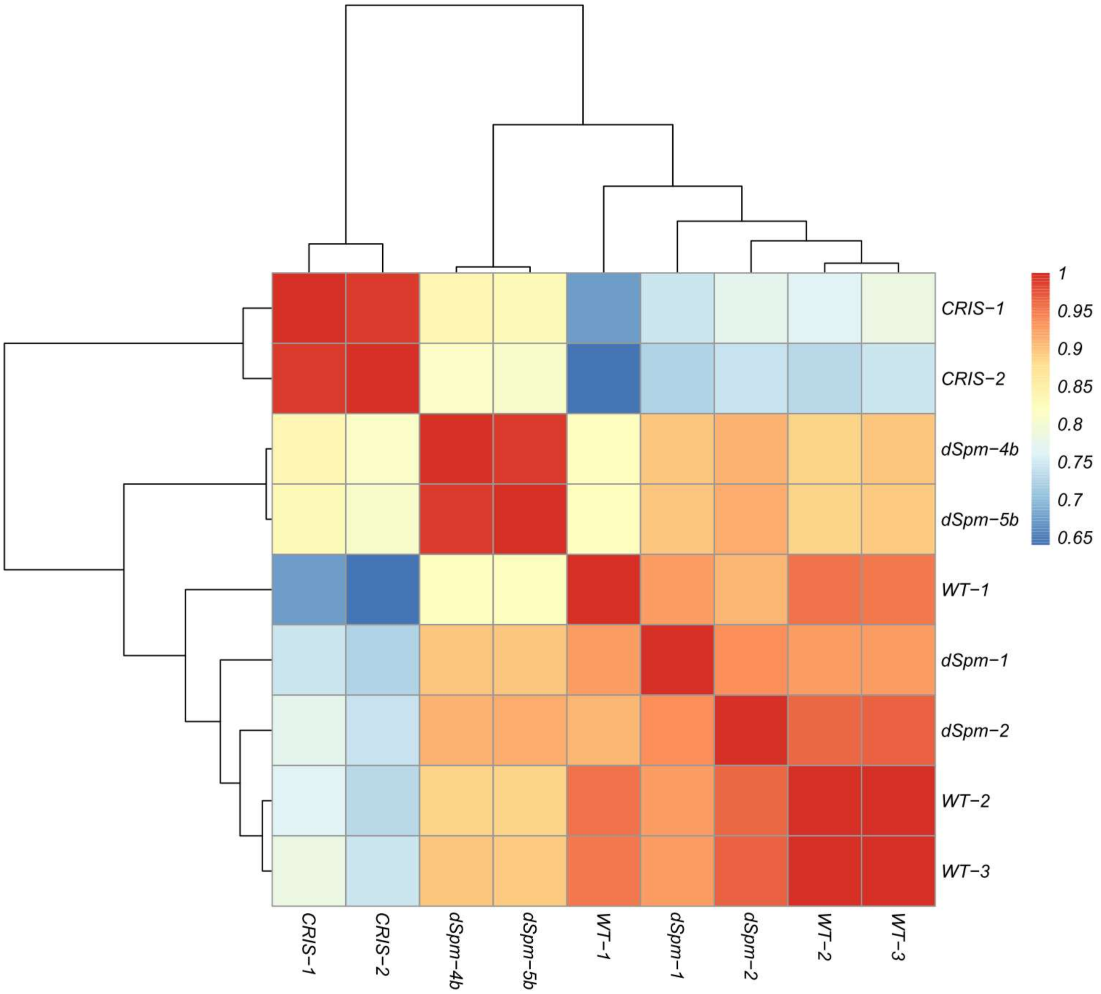

Supplementary Figure S3. Heat map of expression correlation of pairwise samples in RNA-seq data.

The abscissa and ordinate are sample numbers, and their order is determined by the results of sample correlation clustering. On the left is the corresponding cluster tree. The color reflects the magnitude of the correlation between the samples, as shown in the legend on the right.

-

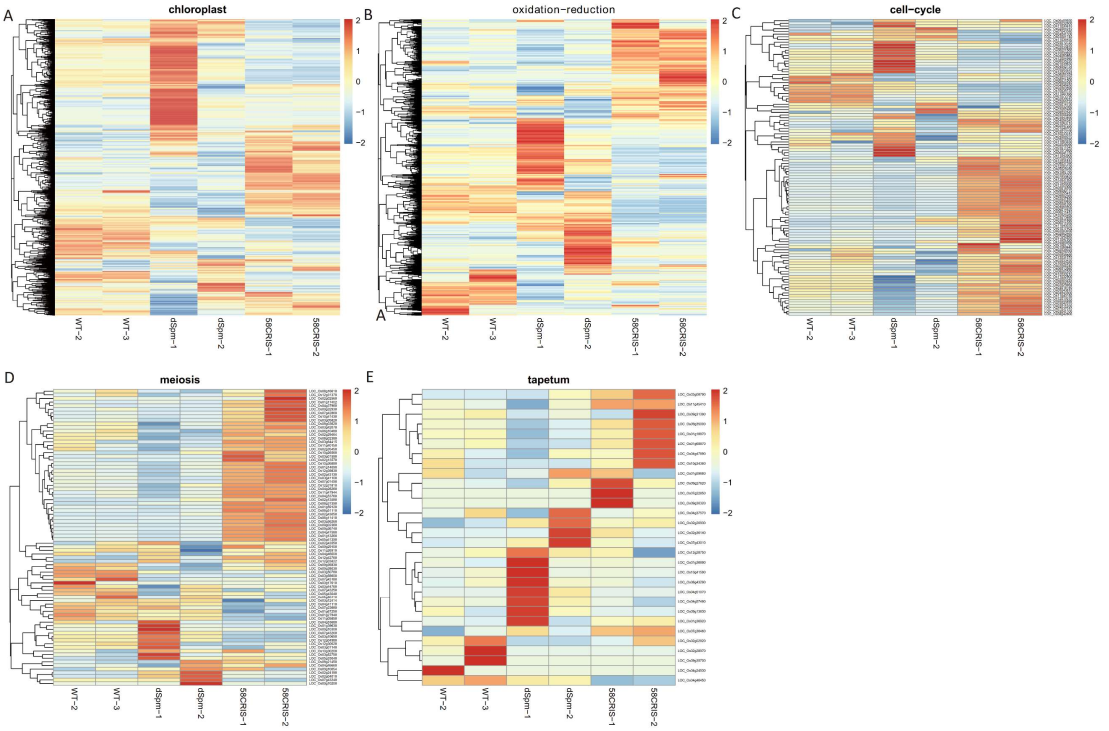

Supplementary Figure S4. Expression profiles of genes with known functions in different samples.

The genes related to chloroplast (A), oxidation-reduction (B), cell cycle (C), meiosis (D), and tapetum (E) were shown.
