# Supplementary figures and images for: OsMADS58 Stabilizes Gene Regulatory Circuits during Rice Stamen Development
Source: Plants (Basel). 2022 Oct 28;11(21):2899. doi: 10.3390/plants11212899 (PMC9658454; doi:10.3390/plants11212899)

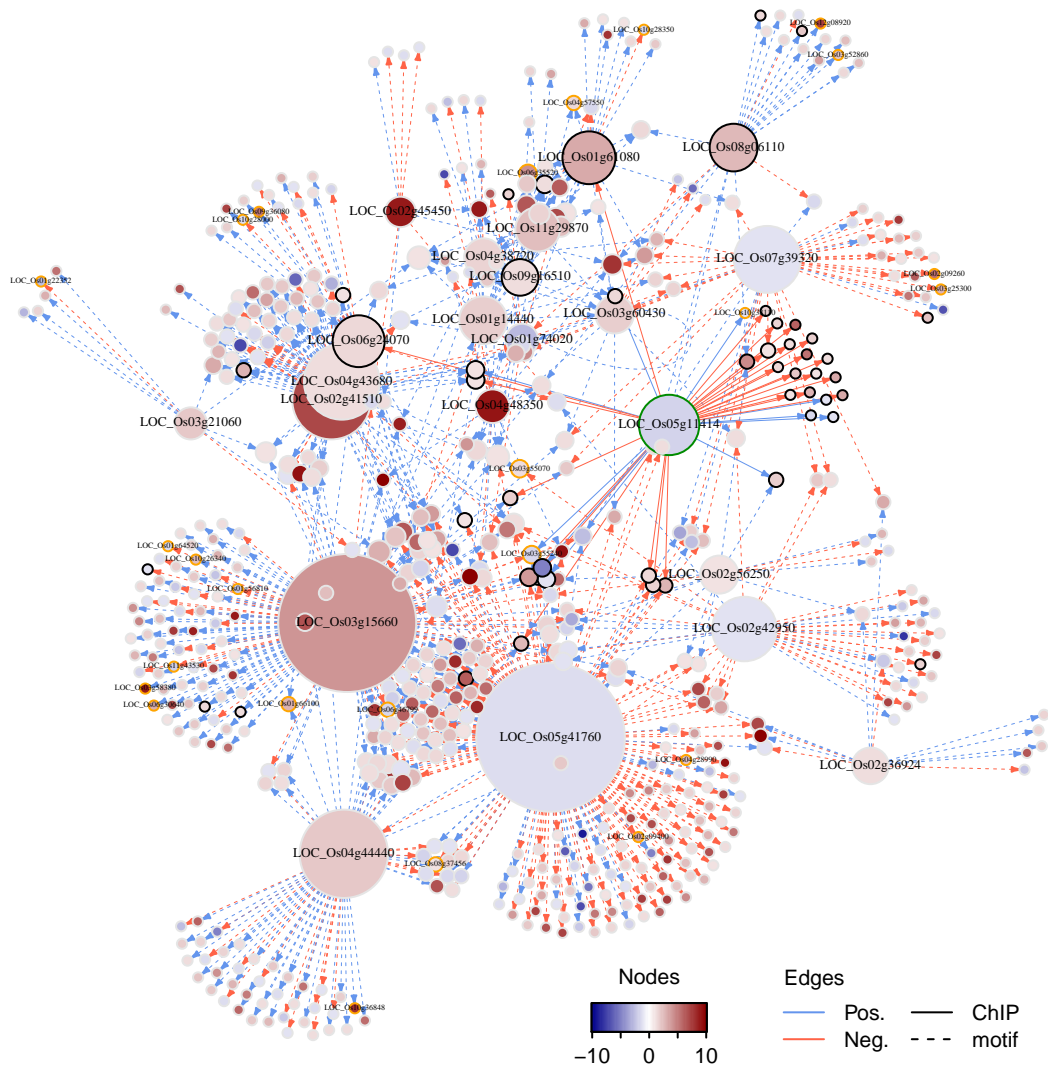

Supplement: Supplementary file 1 [file plants-11-02899-s001.zip › Figure 10A WT dSpm network.pdf]

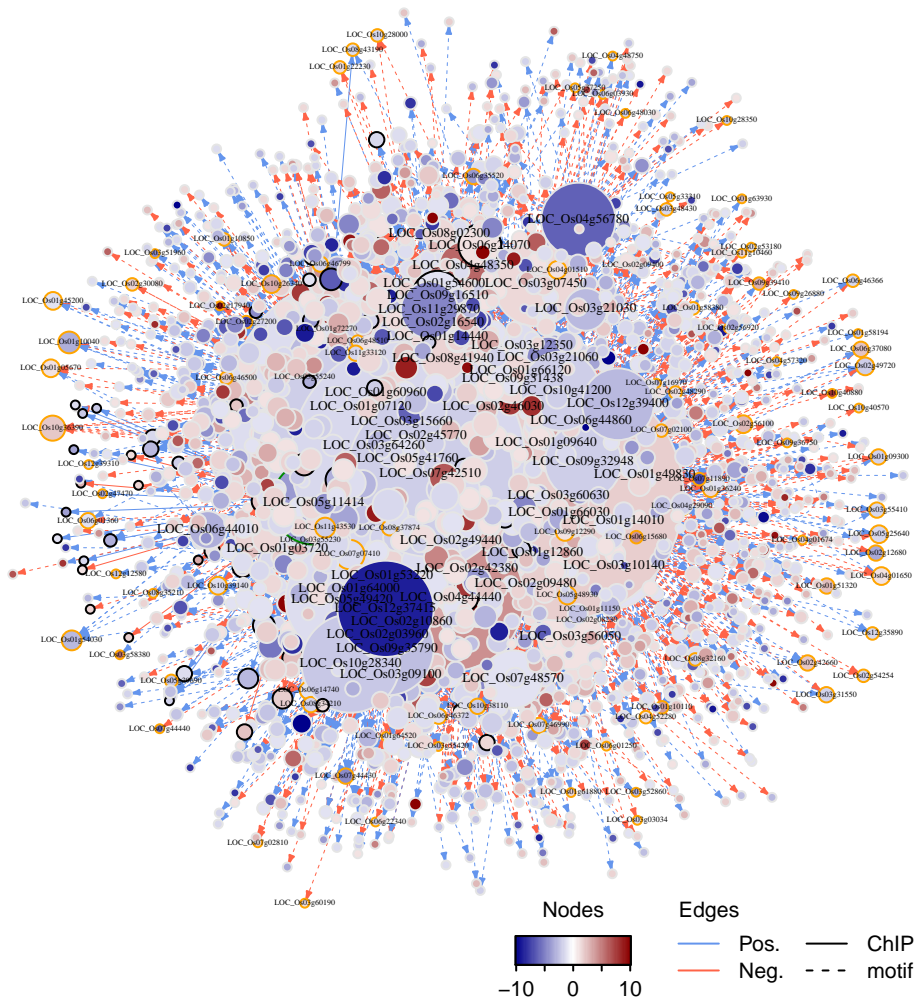

Supplement: Supplementary file 1 [file plants-11-02899-s001.zip › Figure 10B WT CRIS network.pdf]

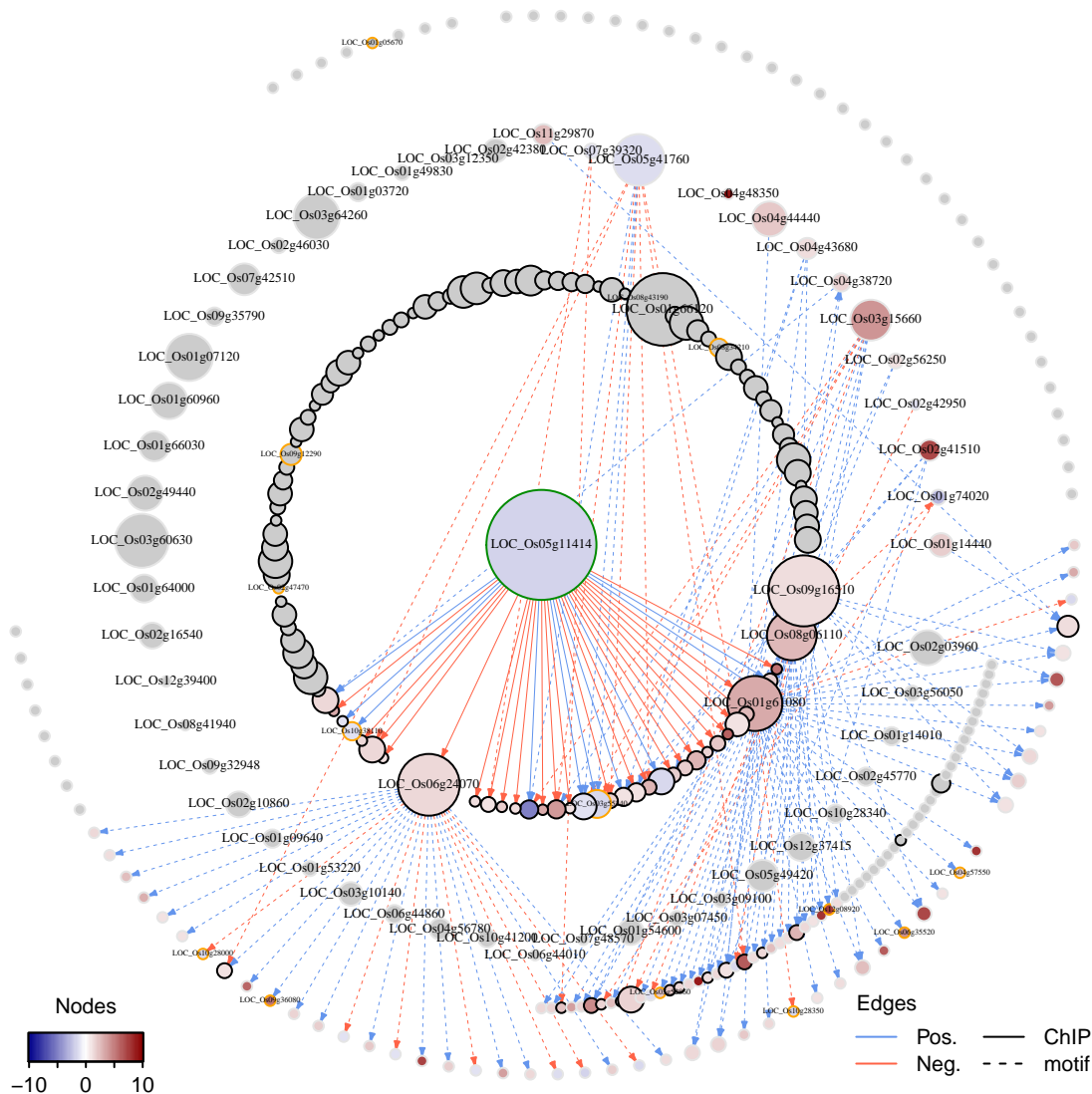

Supplement: Supplementary file 1 [file plants-11-02899-s001.zip › Figure 8A WT dSpm network_sub.pdf]

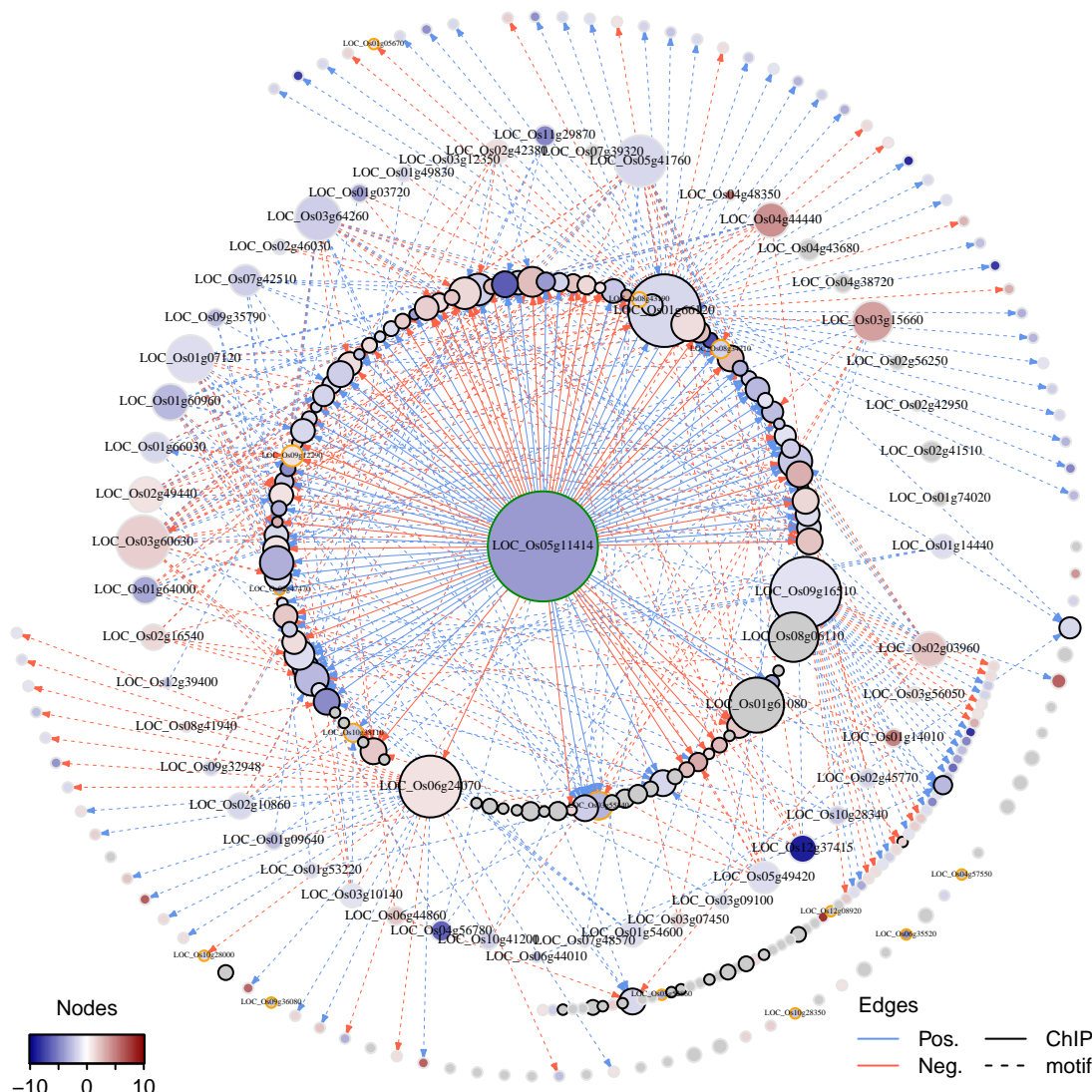

Supplement: Supplementary file 1 [file plants-11-02899-s001.zip › Figure 8B WT CRIS network_sub.pdf]

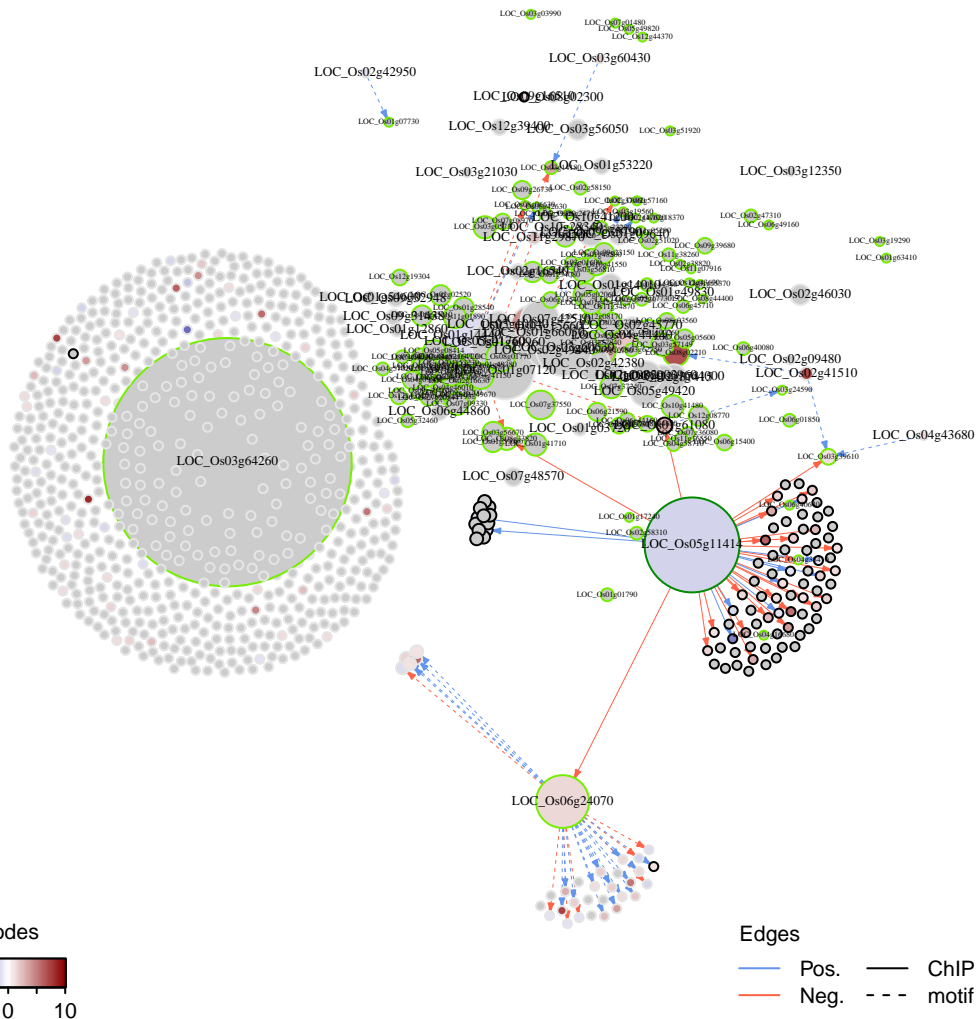

Supplement: Supplementary file 1 [file plants-11-02899-s001.zip › Figure 8C WT dSpm network_sub_chloroplast.pdf]

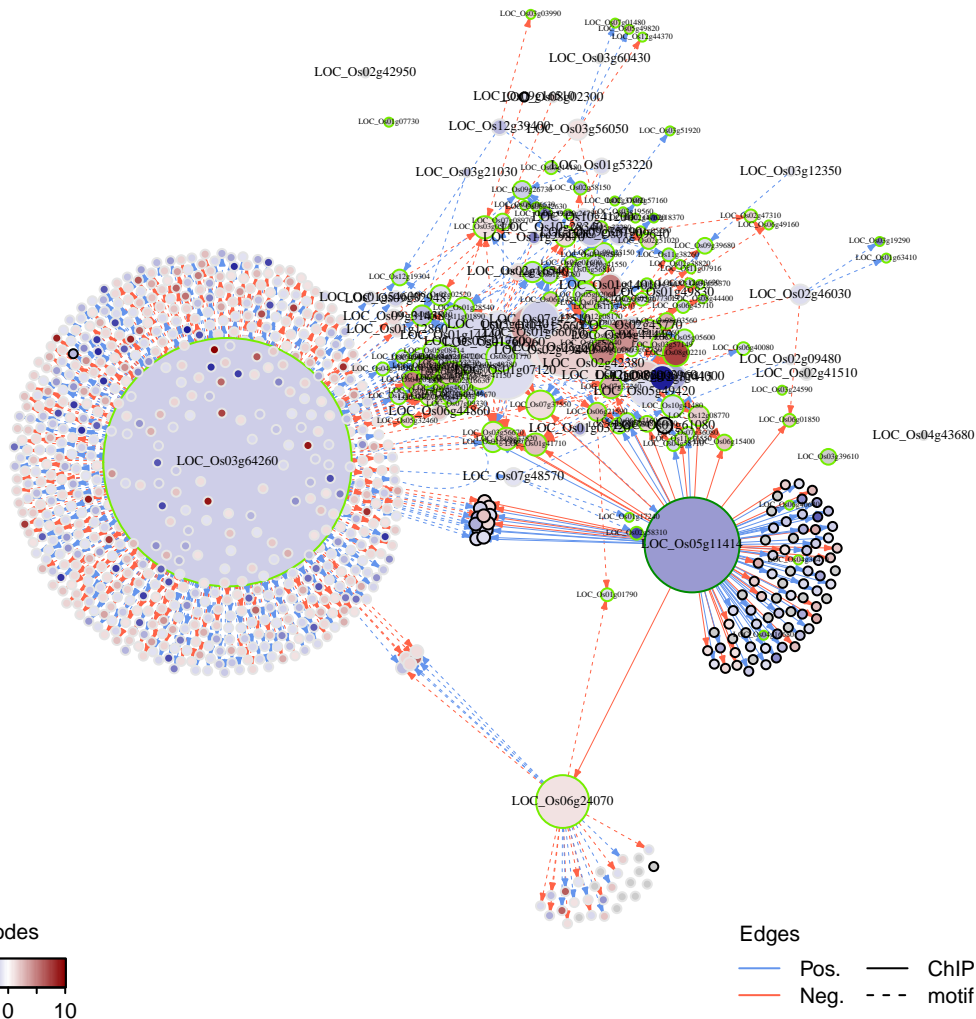

Supplement: Supplementary file 1 [file plants-11-02899-s001.zip › Figure 8D WT CRIS network_sub_chloroplast.pdf]

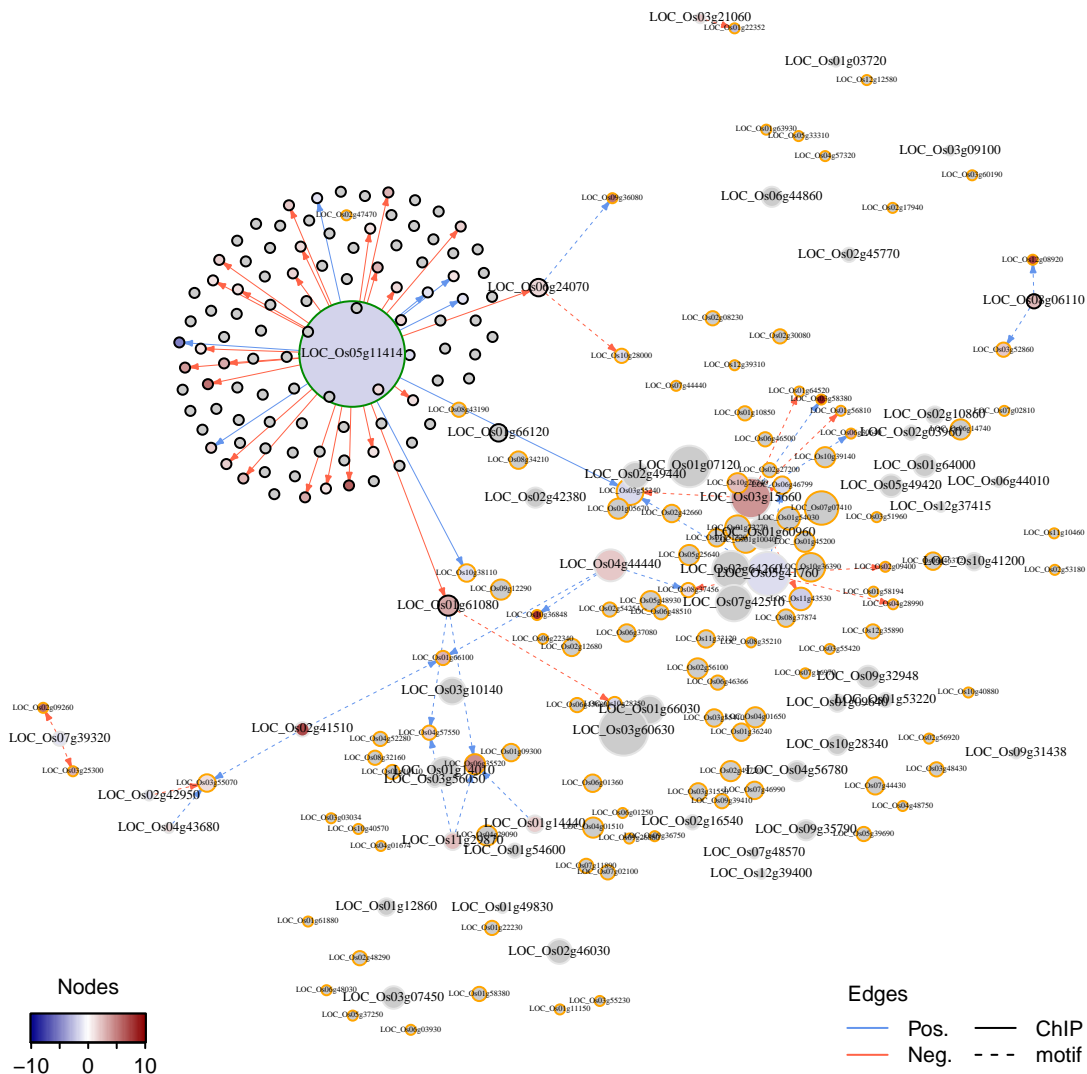

Supplement: Supplementary file 1 [file plants-11-02899-s001.zip › Figure 8E WT dSpm network_sub_oxidation-reduction.pdf]

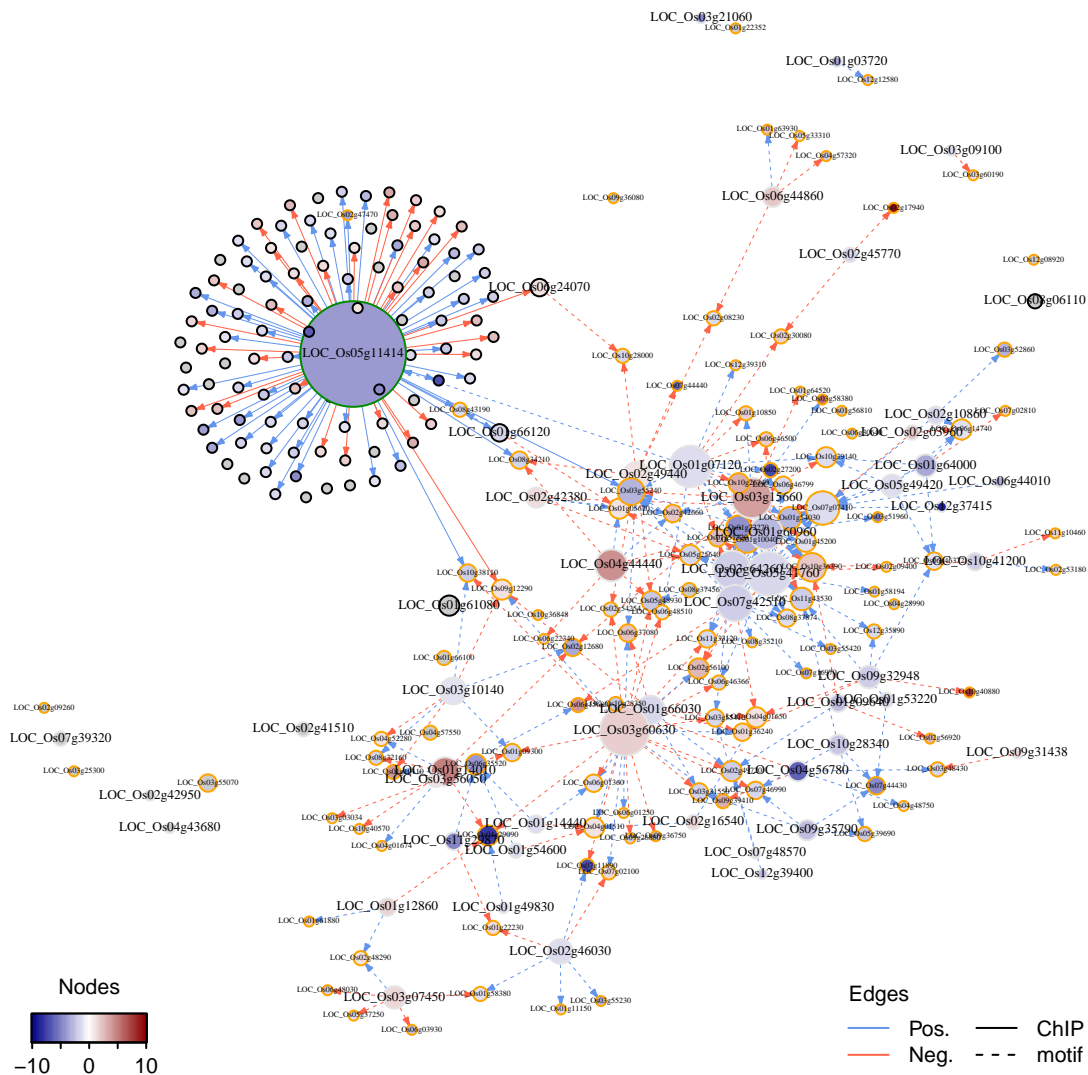

Supplement: Supplementary file 1 [file plants-11-02899-s001.zip › Figure 8F WT CRIS network_sub_oxidation-reduction.pdf]

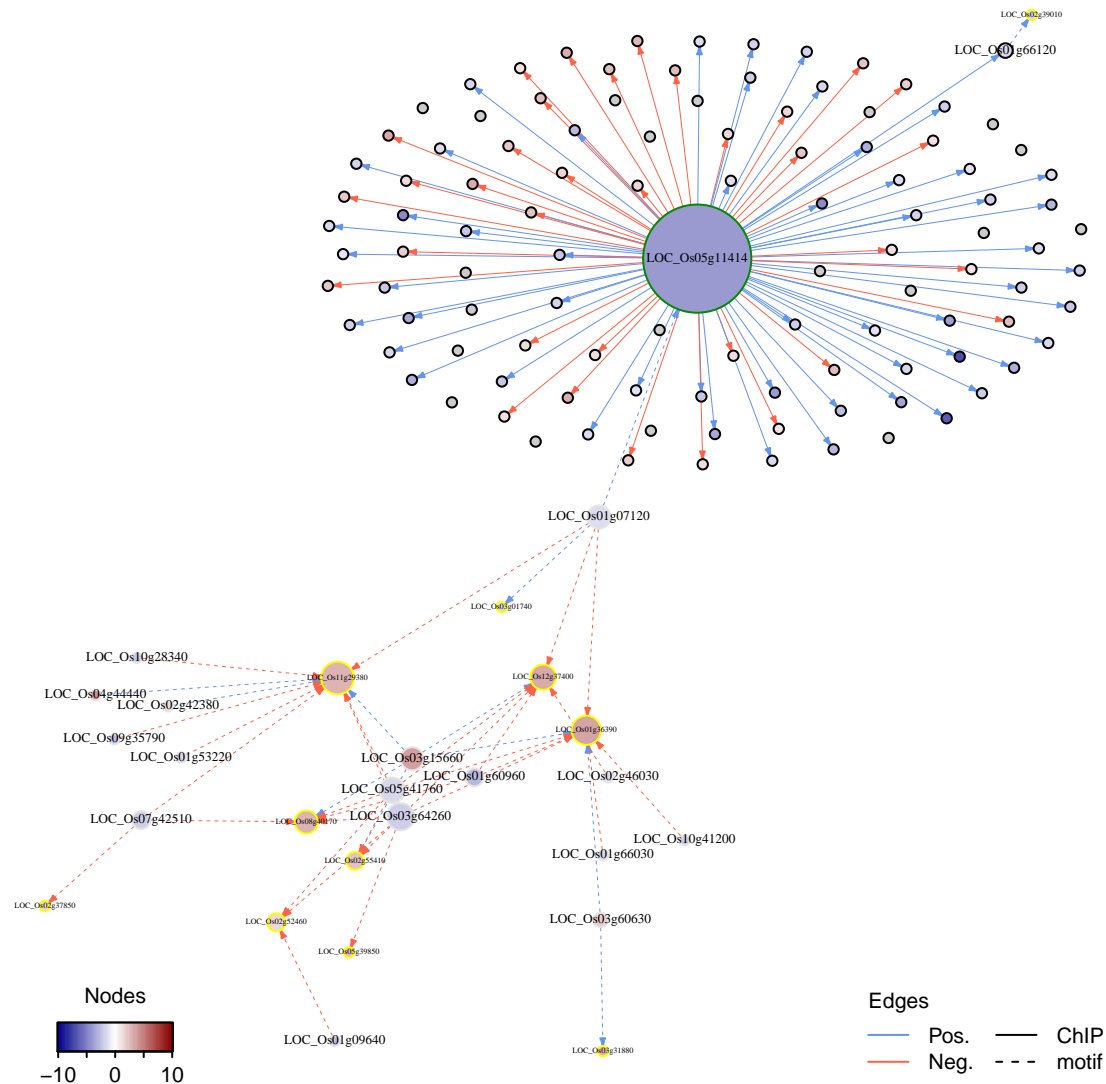

Supplement: Supplementary file 1 [file plants-11-02899-s001.zip › Figure 8H WT CRIS network_sub_cell-cycle.pdf]

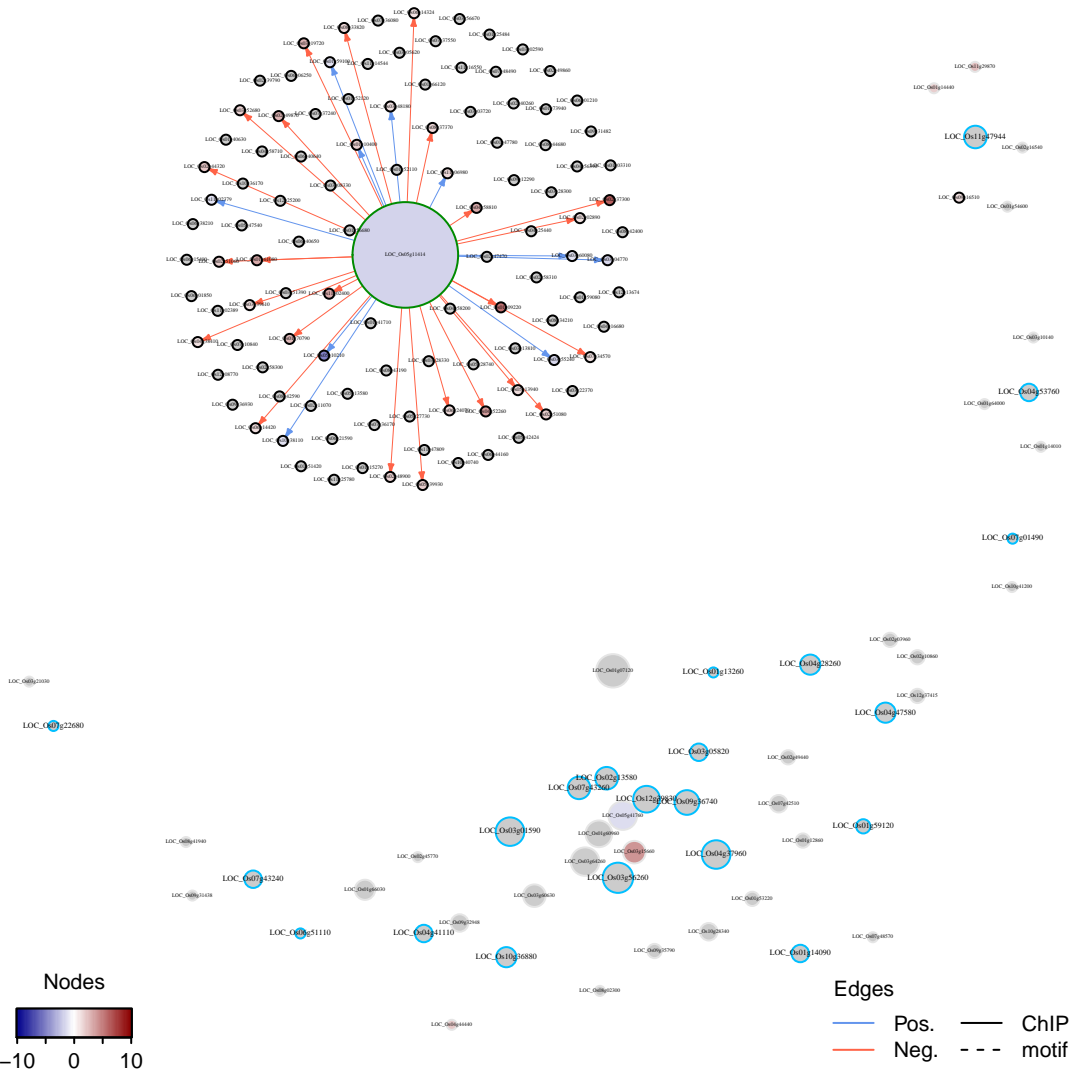

Supplement: Supplementary file 1 [file plants-11-02899-s001.zip › Figure 8I WT dSpm network_sub_meiosis.pdf]

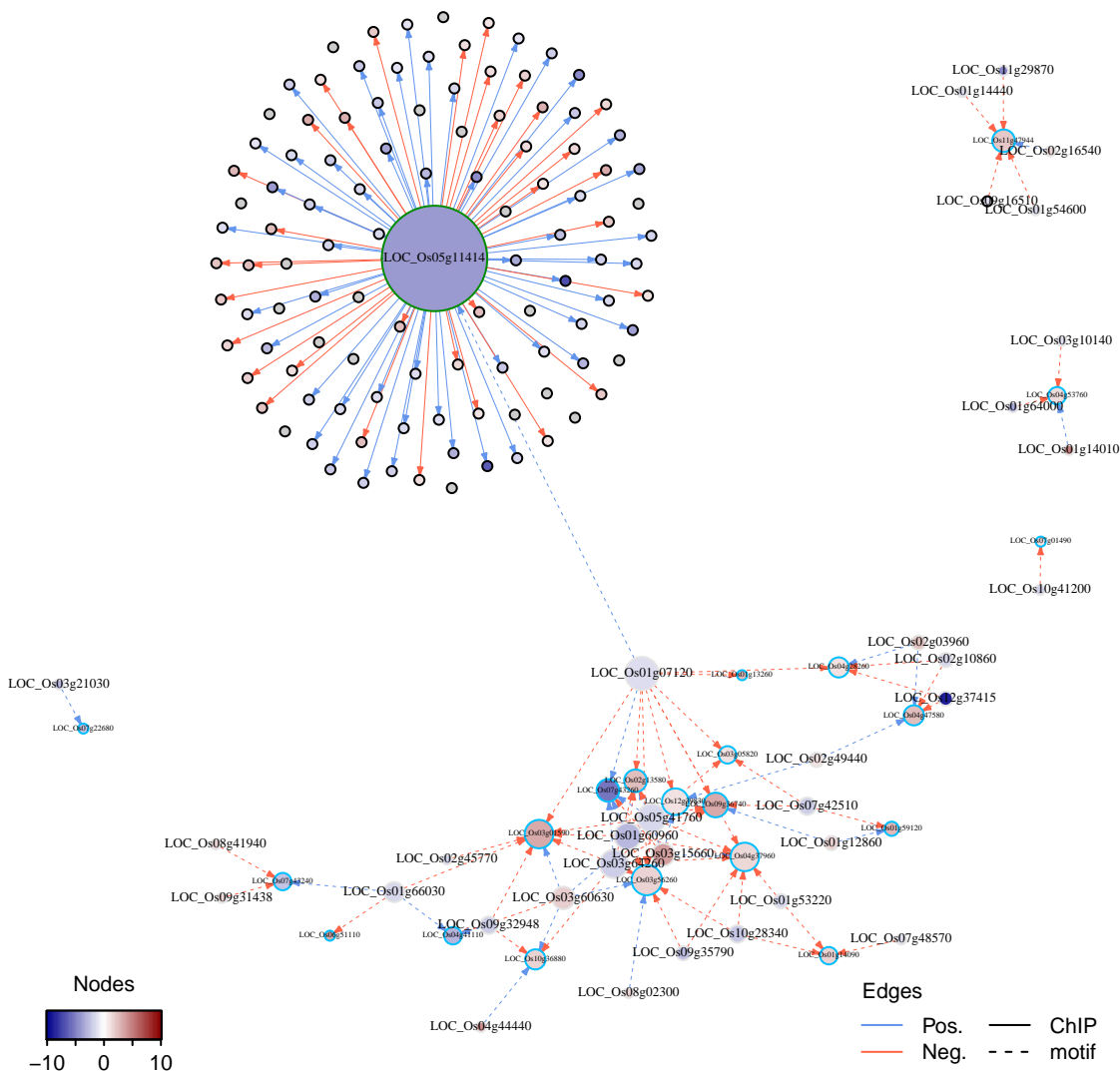

Supplement: Supplementary file 1 [file plants-11-02899-s001.zip › Figure 8J WT CRIS network_sub_meiosis.pdf]

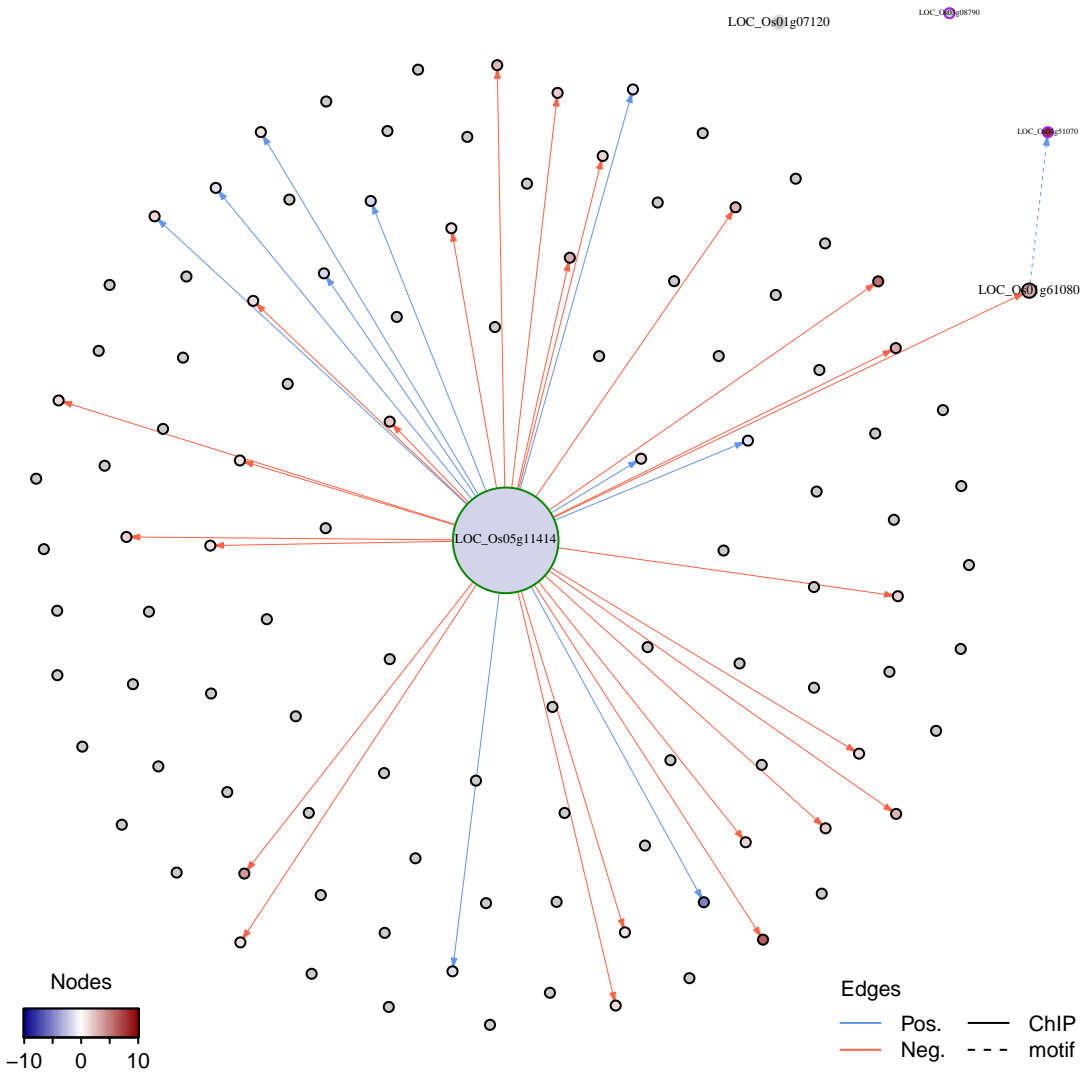

Supplement: Supplementary file 1 [file plants-11-02899-s001.zip › Figure 8K WT dSpm network_sub_tapetum.pdf]

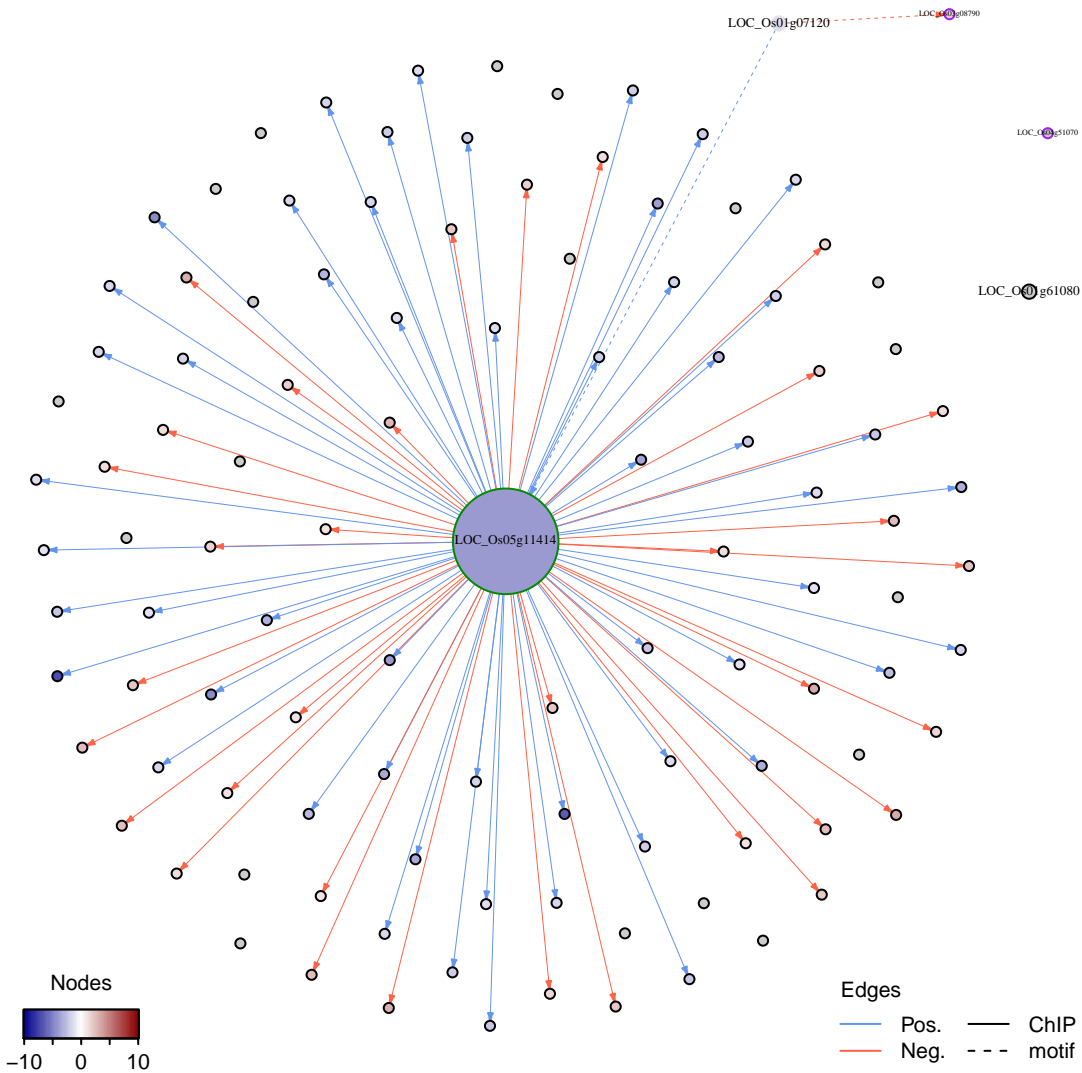

Supplement: Supplementary file 1 [file plants-11-02899-s001.zip › Figure 8L WT CRIS network_sub_tapetum.pdf]
