# Supplementary material for: OsMADS58 Stabilizes Gene Regulatory Circuits during Rice Stamen Development
Source: Plants (Basel). 2022 Oct 28;11(21):2899. doi: 10.3390/plants11212899 (PMC9658454; doi:10.3390/plants11212899)
Supplement: Supplementary file 1 [file Figure 8G WT dSpm network_sub_cell-cycle.pdf › Figure 8G WT dSpm network_sub_cell-cycle.pdf]

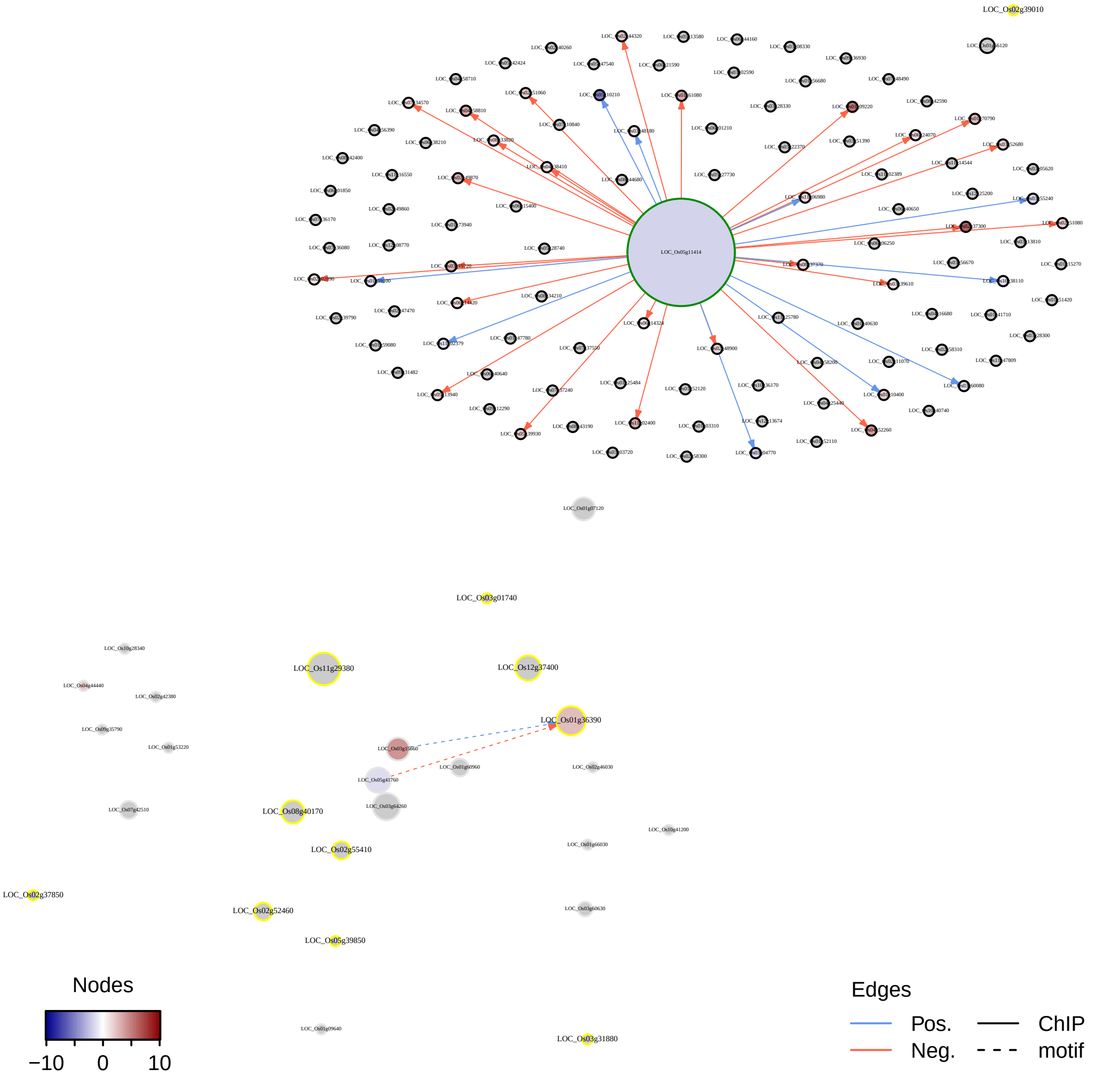
LOC_Os02g39010
LOC_Os05g11414
LOC_Os01g07120
LOC_Os03g01740
LOC_Os10g28340
LOC_Os11g29380
LOC_Os12g37400
LOC_Os04g44440
LOC_Os02g42380
LOC_Os01g36390
LOC_Os09g35790
LOC_Os01g53220
LOC_Os03g15660
LOC_Os01g60960
LOC_Os02g46030
LOC_Os05g41760
LOC_Os07g42510
LOC_Os08g40170
LOC_Os03g64260
LOC_Os10g41200
LOC_Os01g66030
LOC_Os02g55410
LOC_Os02g37850
LOC_Os02g52460
LOC_Os03g60630
LOC_Os05g39850
LOC_Os01g09640
LOC_Os03g31880
Nodes
−10
0
10
Edges
Pos.
Neg.
ChIP
motif
